# Progress in Medicine

**Published:** 1901-01-19

**Authors:** 


### PROGRESS IN MEDICINE.

### RESPIRATORY DISEASES.

**Asthma.**—In a clinical lecture on asthma, Wethered[1] deals both with the true spasmodic and bronchial varieties. The difficulty lies in expiration, and the most marked auscultatory signs are the absence of respiratory murmur, and rhonchi of all sorts, and fascial creaking. Expectoration begins as the attack subsides, and in the spasmodic form epithelial cells, Curschmann's spirals, and Charcot-Leyden crystals are characteristic ingredients. Asthma is often found alternating with brow ague, coryza, and hay fever. It most frequently begins in the first ten years of life, in children coming of a nervous stock. Exciting peripheral causes may be classed as nasal, dental, bronchial, gastro-intestinal, uterine, cutaneous, and pneumogastric. The pathological condition is muscular spasm of the small bronchi, and rapid tumefaction of the mucous membrane of the bronchi. With regard to treatment, most cases do best whilst living in large towns, but in the winter months Bournemouth, Ascot, Aldershot, and Weybridge prove beneficial. Bronchial cases may spend the winter at Torquay or in the South of France, or Mont Dore. The compressed-air treatment, as carried out at Ben Rydding, is helpful, especially to bronchial cases. Diet should be carefully regulated, and the chief meal partaken of at mid-day. The digestive functions and bowels must be regulated. Between the paroxysms arsenic and iodide of potash may be given, or iodide of potash with sal volatile. Three-grain doses of quinine three times a day sometimes ward off paroxysms. In bronchial asthma a mixture of carbonate of soda, iodide of potash, vinum ipecacuanha, with tinctures of lobelia and strammonium is servicable. Acute paroxysms may be relieved by hypodermic injections of morphia ($\frac{1}{6}$gr.) and atropine ($\frac{1}{200}$gr.), or by the inhalation of chloroform or oxygen, the last-named being especially efficacious if there be bronchitis. Caffeine is also useful. Kingscote[2] attributes all true spasmodic asthma to irritation of the vagi or sympathetic. Chief amongst such sources of irritation he places congestion of liver, cardiac dilatatiou, and over-distension of the pulmonary alveoli. The dilated heart, in his opinion, presses on the subjacent vagi and so excites spasm. For this lesion the Schott treatment is effective. The pulmonary distension may be remedied by breathing exercises, the nature of which he gives in detail. Fraenkel[3] has made post-mortem examinations of two cases in which death occurred during a paroxysm of asthma. In one the smaller bronchi showed catarrhal desquamation, the lumen being occluded with compressed ciliated epithelium. In the second case the bronchi were filled with mucus plugs of extraordinary toughness, with Curschman's spirals and with crystals.

**Bronchitis.**—Wilcox[4] gives the following directions for the treatment of senile bronchitis. In acute exacerbations of chronic bronchitis, ammonium carbonate may be given in five or ten grain doses in two or three ounces of milk, and during convalescence or in the chronic stage one-fortieth to one-twentieth of a grain of strychnine sulphate every three or six hours. To disinfect the expectoration creosote carbonate is effective in twenty-drop doses given in two ounces of sherry every four hours. Massage of the chest wall affords great relief, and the atmosphere of the room may be artificially dried by the exposure of calcium chloride or strong sulphuric acid in shallow earthen receptacles. Opium in any form is contra-indicated.

A fatal case of plastic bronchitis is reported by Vintras.[5] The patient, a man of sixty-eight years, complained of cough, shortness of breath, hæmoptysis, and pains in the chest. On auscultation rhoncai and sonorous and sibillant râles were heard all over the chest. There was emphysema, and some dulness at the right base. Twice whilst under observation the patient was seized with intense dyspnœa, incessant, harassing cough, and lividity. When suffocation appeared imminent, relief was afforded by the expectoration of rounded, dark, red masses which proved to be fibrinous casts of the bronchi. The third attack proved fatal. At the necropsy a malignant growth of the œsophagus involving the trachea was found, a cast blocked the right bronchus which was intensely congested, and other casts were also found in the tubes of the right middle lobe. The pleuræ were adherent, the heart enlarged. There were no tubercles. Plastic or fibrinous bronchitis is a rare disease, only about one hundred cases being recorded.

**Pneumonia.**—The question of the infectivity of pneumonia is discussed by Dingle in the annual medical report of Middlesbrough for the year 1899. During the year a second epidemic of this disease visited the town, and it is calculated that there were 1,126 cases. Twenty-five per cent. of the cases had been in close contact with recent cases, and it appeared that infection might lie dormant in a house for a considerable length of time. Infection could not be traced to food, but the incidence was heavy in houses having privy-middens. Handford[6] is confident that under good sanitary conditions the risk of infection is infinitesimal, except in influenza. He records a series of six cases occurring in one family, which were probably of this nature, and a series of twenty-eight cases and eight deaths in a small village. In prisons, workhouses, and ships, and in insanitary buildings outbreaks of pneumonia are not uncommon. Other forms of infectious pneumonia are seen in plague, typhoid fever, and food poisonings. Commenting upon the varieties of the disease the same writer instances cases in which, with well-marked physical signs, the temperature did not rise above 100° Fahr., and others in which, although the symptoms were well marked, the physical signs of consolidation were absent throughout the illness. The symptoms may precede the physical signs by several days. Probably the great diversity of type seen in this disease is due to different varieties of organisms which may act as exciting causes.

In cases of pneumonic consolidation running a very chronic course resolution may be sometimes brought about by exploratory puncture with an aspirating needle. Macalister and Bell[7] have recently placed on record two successful cases. Occasionally such cases go on to fibrosis and induration; recent researches by Ribbert go to show that the connective tissue formed arises not from the alveolar walls, or subpleural and interlobular tissue, but from the connective tissue in the wall of the smallest bronchi and bronchioles, which is highly vascular.

The subject of hydrotherapy in connection with pneumonia has attracted considerable attention of late. At the German Congress of Medicine, Nothnagel and Senator both advocated this line of treatment. Hayem[8] in critical cases uses the cold bath (68° Fahr.) for ten minutes if the temperature be above 103° Fahr., repeating the process every three, four or six hours if necessary. Marfau employs the same method for children when there is hyperpyrexia, stupor or delirium. The temperature of the bath varies from 83° to 96° Fahr., the child being immersed for ten minutes every three hours. By this means he claims that the pulmonary congestion is lessened, the heart strengthened, urine increased, and nervous symptoms quieted. Baruch[9] whilst approving of the bath for infants, prefers in the case of adults to apply compresses of cold water (60° Fahr.) extending from the clavicles to the umbilicus. The compress completely encircles the trunk, being slit at either axilla. It is covered by one layer of flannel and renewed every hour, whilst the rectal temperature remains above 100° Fahr. By this means a valuable stimulant effect and counter-irritation are obtained. In addition to the compresses he advises the systematic administration of four ounces of iced-water every two hours, for purposes of renal stimulation. Laudouzy[10] also supports these hydro-therapeutic measures in hyperthermic and adynamic cases of pneumonia. Mays[11] prefers to use large flat ice-bags to the head and chest. Ewart[12] has tried the subcutaneous injection of normal saline (NaCl. ʒi to a pint) in pneumonia, as recommended by Penrose. About a pint is administered daily until the crisis. His results were disappointing.

**Cheyne-Stokes Respiration.**—Pembrey[13] points out that this form of respiration is not diagnostic of any disease, but that it may be and has been observed under a large variety of conditions. Thus it is seen during hibernation in dormice and bats, and in healthy adults and children during sleep. It may also be induced in human subjects by certain drugs such as chloral, morphine, chloroform, ether, and potassium bromide. The explanation of the phenomenon is to be sought for not in disturbance of the vascular system, but in altered irritability of the cells of the central nervous system. It is unwise to base a prognosis simply upon the existence of this sign.

**Embolism of the Pulmonary Artery.**—A case of pulmonary embolism is recorded by Drasche.[14] The symptoms were as follows:—Syncope and loss of consciousness, restlessness, jactitation, dyspnœa, a small, irregular pulse, cyanosis and cold perspirations. There was a humming thrill over the second and third intercostal spaces just outside the sternum, and rumbling murmurs throughout the whole cardiacycle. A site from which the thrombus might have originated should be sought for.

**Therapeutics.**—Cacodylic acid is recommended by Ewert[15] as a remedy in phthisis. Cacodyl is a dimethyl arsenide of unpleasant taste and smell, but the soda salt is free from these objectionable features, and contains 46 per cent. of arsenic. It may be administered by the mouth, or, better, by the rectum, or subcutaneously. The dose by the mouth is half a grain three or four times daily, and in other methods of medicature this may gradually be increased to a grain. The salt is freely soluble, and large quantities of arsenic may thus be introduced into the system without giving rise to poisonous effects. Hectic fever is diminished, cough and expectoration lessened, and the general nutrition improved. Gautier[16] attributes the therapeutic value of the drug to the induced hyperleucocytosis, the increased number of red corpuscles and augmented oxidation. Kakodylic elixir and kakodylic globules are convenient forms of medication. Landerer's treatment of phthisis by sodium cinnamate has been very widely adopted, more particularly in Germany. The drug in solution is injected intravenously, the initial dose of 1 mgm. being gradually increased up to 15 mgm. Hessen,[17] of Mannheim, speaks highly of his results even in advanced cases, but Ewald's[18] report is not so sanguine. Sodium cinnamate ("Hetol") acts by causing inflammation and cicatrisation around the tuberculous foci. Krompecher[19] conducted a series of experiments on animals, first injecting cinnamate of sodium, and, some days later, pure cultures of tubercle bacilli. In all cases the animals died of tuberculosis. Animals first infected with tubercle and then subjected to the cinnamate of soda injections, died as rapidly as the control animals to whom the drug was not administered.

[1] Clin. Jour., May 30, 1900. [2] Brit. Med. Jour., Oct. 15, 1900. [3] Dent. Med. Woch., Nov. 17, 1900. [4] Amer. Jour. of Med. Sci., cxix., Nov. 5. [5] Lancet, Sept. 15, 1900. [6] Lancet, July 21, 1900. [7] Lancet, Nov. 3, 1900. [8] Practitioner, May 1900. [9] Med. Rec., Aug. 4, 1900. [10] Rev. de Therap., 57, page 433. [11] Merck's Arch., May 1899. [12] Brit. Med. Jour., Sept. 29, 1900. [13] Brit. Med. Jour., Sept. 30, 1899. [14] Wien. Klin. Woch, June 7, 1900. [15] Lancet, March 10, 1900. [16] Rev. de Therap., No. 13, p. 433. [17] Therap., March 15, 1900. [18] Berlin Klin. Woch., May 21, 1900. [19] An. de l'Inst. Past., Nov. 25, 1900.

